# Study on the effects of the shot peening intensity on the microstructure, friction and wear properties of high-strength steel

**DOI:** 10.1371/journal.pone.0314561

**Published:** 2024-12-19

**Authors:** Huashen Guan, Junjie Zhang, Xiaoguang Zhang, Guofu Sun, Chenfeng Duan

**Affiliations:** 1 Jiangmen Power Supply Bureau of Guangdong Power Grid Co. Ltd., Jiangmen, Guangdong, China; 2 School of Mechanical and Automotive Engineering, South China University of Technology, Guangzhou, China; Gazi University: Gazi Universitesi, TÜRKIYE

## Abstract

The microstructure, hardness, residual stress, and friction and wear properties of 25CrNi2MoV steel with different particle diameters during shot peening strengthening were studied. Studies have shown that a grain refinement layer appeared on the surface of the material after shot peening. The shot peening intensity increased with increasing particle diameter; a greater shot peening intensity corresponded to a greater surface hardness of the material, the maximum hardness was 592 HV_0.2_, and the residual compressive stress on the material surface was 725 MPa. A shot peening finite element model was established to accurately predict the residual stresses in the samples after shot peening. The prediction errors were 1.4–7.9%. The finite element model indicates that the maximum residual stress occurs in the subsurface layer. After shot peening, the wear resistance of the sample significantly improved, and the amount of wear significantly decreased. Therefore, shot peening can significantly improve the mechanical properties and wear resistance of high-strength steel, which increases the service life of parts.

## 1. Introduction

With the rapid development of the machinery industry, the performance requirements for industrial parts are increasing. The gearbox drive shaft is an important component for transmitting power. In particular, when the transmission shaft is transmitting power, it experiences considerable alternating stress, contact stress, strong impact and friction [1] and is prone to failure. Therefore, improving the wear resistance of a transmission shaft can significantly improve the working conditions and service life of the shaft.

Surface strengthening technology can increase the surface hardness of a part, improve the surface micromorphology, and introduce a depth of residual stress field to the surface of the part, which significantly improves its anti-wear and fatigue performance. Generally, surface strengthening technology strengthens the surface of parts through one or more surface-strengthening treatment processes, which mainly include surface quenching technology [2], surface rolling technology [3] and shot peening strengthening technology [4, 5]. Xie et al. [6] used the strengthened grinding process (SGP) to strengthen the surface of a weld seam of 12Cr17Mn6Ni5N steel. The results showed that the surface hardness increased by 109.9% compared to that of the untreated sample, and the residual stress increased from 46.5 MPa to 1253.1 MPa. On this basis, Xiao et al. [7] introduced ultrasonic shock into SGP (USGP) and reported that compared with untreated samples, the wear of samples treated with USGP reduced the friction coefficient by 38% and 48.96%, respectively. Xie et al. [8] treated GCr15 steel balls with mechanical alloying and NH_3_·H_2_O strengthening. The results showed that the microhardness of the sample surface was 895 HV, and significant grain refinement and high dislocation density were observed.

Compared with the above technology, shot peening technology is advantageous because it is not affected by the length and shape of the parts to be processed and it has a short strengthening period, an obvious strengthening effect, a low cost, and it is easy to operate. Shot peening can form a high-density dislocation structure in the strengthening layer on the material surface and refine the grains. Moreover, shot peening can generate a depth of residual stress in the surface strengthening layer of a material. The combined action of the two can improve the fatigue resistance, wear resistance and stress corrosion properties of the material [9–11]. Gerin B et al. [12] conducted shot peening on C70 steel and reported that the shot peening process introduced important residual compressive stress and microstructure gradients on the material surface. The appearance of residual compressive stress enhances the fatigue resistance of the material. Zhang J et al. [13] reported that traditional shot peening treatment could increase the surface roughness of materials, whereas particle impact could reduce the surface roughness of materials. The material surface exhibited significant morphological changes from grinding grooves to small craters.

Currently, finite element modeling of shot peening can realistically simulate the dynamic process of shot peening reinforcement. Finite element simulation can obtain performance indicators such as the residual stress distribution and surface morphology of the samples. Kim et al. [14] established a single-shot grain model to elucidate the formation of residual stress in the surface layer of samples. However, because the mutual influence between projectiles is neglected, this model fails to reflect the impact of coverage on residual stress. Majzoobi et al. [15] developed a multi-grain axisymmetric shot peening model and investigated the impact of parameters such as the projectile velocity, projectile diameter, coverage, and material properties on the magnitude and distribution of residual stress. Nevertheless, the drawback of this model lies in the predetermined position of projectiles that impact the sample, so the dynamic process of projectiles that randomly impact the surface during the actual shot peening process is challenging to accurately simulate. Therefore, there is an urgent need to optimize the current shot peening finite element model to more accurately simulate the actual shot peening process.

Many scholars have shown that shot peening can effectively improve the wear resistance of materials but hold different views on the increase and decrease of the friction coefficient. Menezes M R et al. [16] reported that after shot peening, the wear resistance of austenitic AISI 316L steel improved because a hardened layer formed on the surface. Han X et al. [17] discussed the effect of shot peening on the friction and wear resistance of materials, and the results showed that the wear of AISI5160 steel decreased by 73%. Kovacı H et al. [4] found that after shot peening, AISI4140 steel showed a larger friction coefficient, a smaller wear amount, and enhanced wear resistance. However, some scholars believe that shot peening can reduce the friction coefficient of materials. Mitrovic S et al. [18] showed that shot peening could reduce the friction coefficient and wear rate of materials in both dry and lubricated conditions. Wiratkapun et al. [19] studied the effect of shot peening on the wear properties of 316L stainless steel. The results showed that the hardness of the shot peened sample increased by 126% compared to the untreated sample, and the wear performance of the shot peened sample significantly improved under low-load conditions. This improvement can be attributed to the surface strengthening enhancement via the evolution of micro nano structures. In addition, Fang et al. [20] investigated the effects of laser peening on the wear properties of a GH4169 high-temperature alloy. The results revealed that the wear rate of the sample decreased by 58.6% after laser peening because the hardened layer formed by laser peening could prevent the formation of debris and improve the tangential stiffness of the material.

In this work, a shot peening experiment and a series of performance tests were performed on the transmission shaft material. The effects of pellets with different diameters on the microstructure and wear resistance of the material were studied.

## 2. Experimental procedure and finite element analysis

### 2.1 Experimental procedure

The raw material in the test was 25CrNi2MoV steel, and Table 1 shows the chemical composition. The raw material was processed into a sample of φ 48 mm × 6 mm, and the material was heat treated before the shot peening test. The material was quenched at 860°C for 30 min, oil-cooled to room temperature, tempered at 180°C for 2 h, and finally air-cooled to room temperature.

**Table 1:** Chemical compositions of the 25CrNi2MoV steel (wt. %).

C	Si	Mn	Cr	Ni	Mo	Al	Cu	N	V	Fe
0.26	0.26	0.66	1.55	2.10	0.3	0.097	0.03	0.0222	0.11	Bal

The samples were shot-peened by a pressure feeding automatic shot peening machine (LSWPC1010FK-A). The working principle is to generate power through compressed air and spray pellets to the surface of the workpiece through the nozzle to cause severe plastic deformation on the surface of the workpiece and increase the surface hardness and residual compressive stress of the material. The shot was a cast steel shot. The shot peening intensity of the sample surface in the shot peening process was measured by an arc height measuring instrument (LTSP-3). The test piece was a standard ALMEN test piece. In the shot peening experiment, the shot peening air pressure was 0.4 MPa, and the coverage rate was 200%. Table 2 shows the shot peening process parameters.

**Table 2:** Shot peening process parameters.

Samples	Shot diameter (mm)	Intensity (mmA)
SP1	0.4	0.326
SP2	0.6	0.385
SP3	0.8	0.473

**Fig 1:**
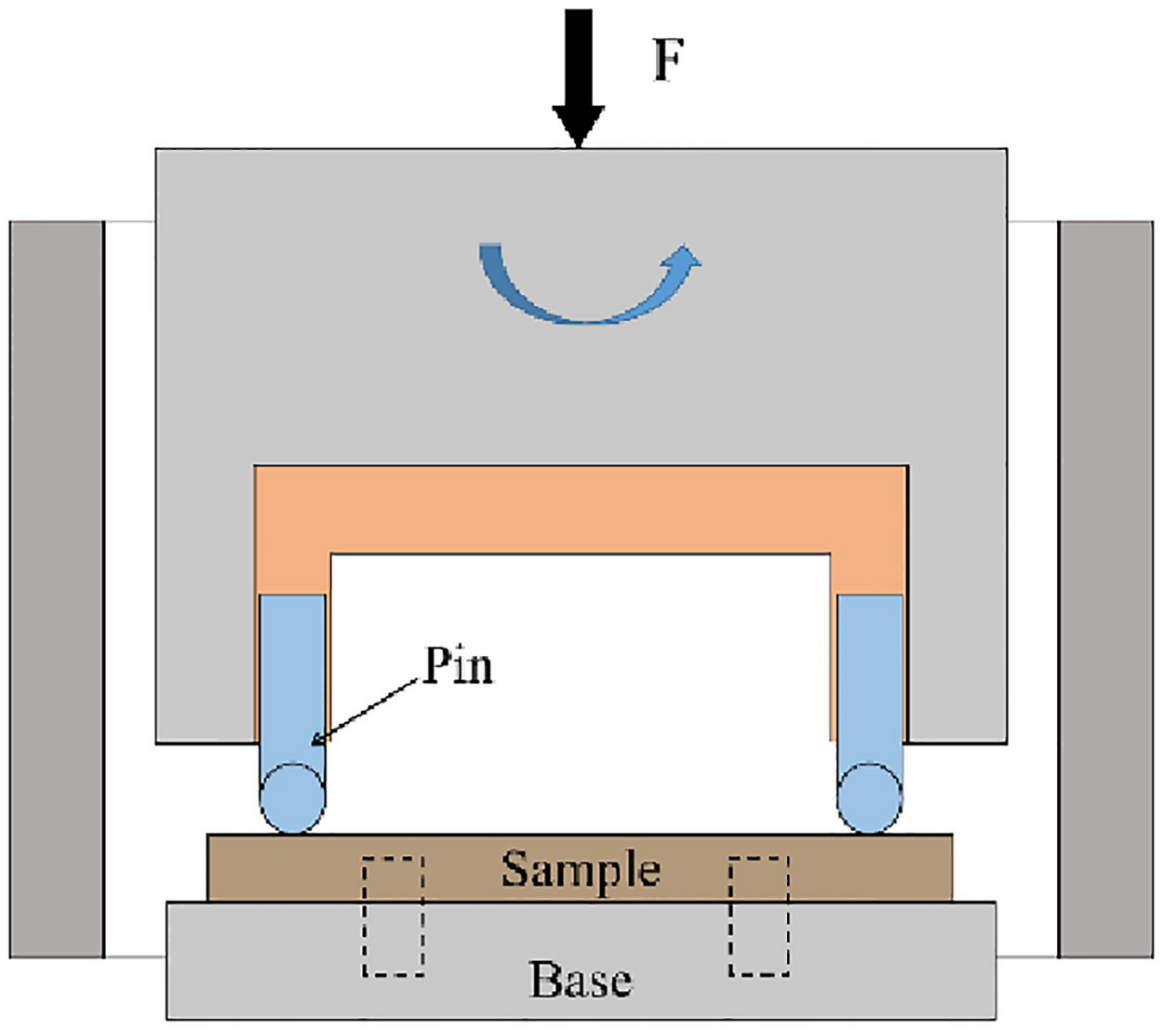
Schematic diagram of pin-disk wear.

A sliding wear test machine (MMU-10G) was used to perform the sliding wear test on the sample, and Fig 1 shows the schematic diagram. This test adopted the pin-pack contact form. The disc sample before and after shot peening was fixed at the bottom. The ball pin was installed at the top and rotated clockwise under the drive of the motor. The ball stud material in this test was GCr15. After the surface had been ground and polished, the surface roughness was 0.3 μm, and the hardness was 60 HRC. Before and after the test, all ball stud and disc samples were placed into an alcohol solution for ultrasonic cleaning and subsequently blown dry. For the sliding wear test, the oil lubrication method was adopted, and the lubricating oil was Mobil DTE10 Excel150 anti-wear hydraulic oil. Table 3 shows the sliding wear test parameters.

**Table 3:** Parameters of the sliding wear test.

Radial load (N)	Spindle speed (r/min)	Operation hours (min)	Lubrication state
150	60	30	Oil lubrication

After the sample had been ground and polished with sandpaper, it was corroded with a 4% nitric acid alcohol solution. Then, the metallographic structure of the sample cross-section was observed with an optical microscope (Leica-M165C). A 3D topography instrument (RTEC UP DUAL-MODE) was used to test the surface 3D topography of the untreated (UT) sample and shot-peened (SP) sample. The scanning area was 2 mm long and 1.2 mm wide. A roughness meter (MARSURF-M300C) was used to measure the surface roughness of the UT sample and SP sample. A Vickers microhardness tester (SCTMC-HV50) was used to test the cross-sectional hardness distribution of the sample, the test load was 200 g, and the loading and holding time was 15 s. The surface residual compressive stress of the sample was detected using an X-ray stress analyzer (PROTO-LXRD). The tube voltage was 25 kV, the tube current was 5 mA, and the Cr target Ka was used for radiation. An environmental scanning electron microscope (Quanta200) was used to observe the sliding wear morphologies of the UT sample and shot-peened sample.

### 2.2 Finite element setup

**Fig 2:**
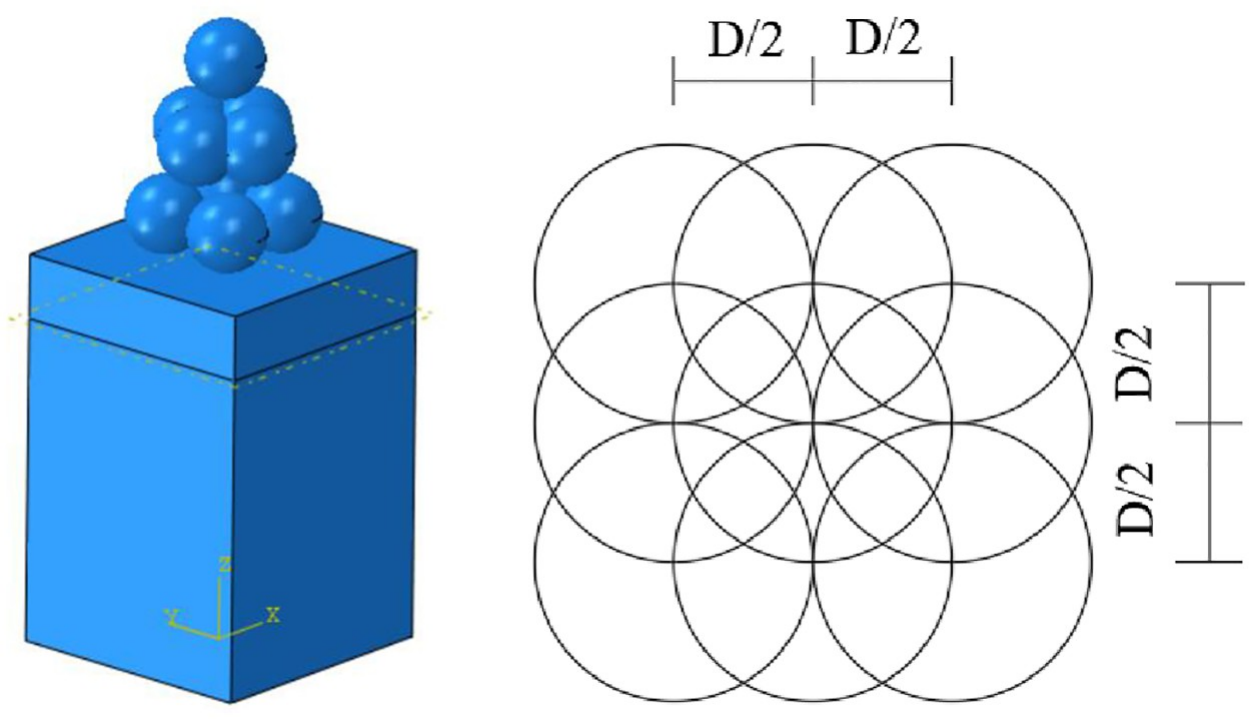
Finite element model of shot peening.

The shot peening finite element model was established using the ABAQUS software. To avoid computational inefficiency, the dimensions of the target material model were set to 2×2×3 mm. In total, 9 projectiles (4+4+1) were included in the finite element model. The first layer consisted of 4 projectiles; the second layer also had 4 projectiles but with a 90° rotation relative to the first layer; the third layer had one projectile positioned in the middle of the sample. This configuration, as illustrated in Fig 2, ensured that the projectiles did not repeatedly impact the same location to achieve a coverage rate of 100%. For the present shot peening simulation, this configuration was applied twice. The impact angle of the projectiles was set at 90°, and the impact velocity was 40 m/s. The projectile diameters in this study were 0.4 mm, 0.6 mm, 0.8 mm, and 1.0 mm. The friction coefficient between the projectiles and the sample was set to 0.2. Table 4 shows the material parameters for both projectiles and samples. The classical Johnson‒Cook model was used as the constitutive model for material behavior. The C3D8R hexahedral linear reduced integral element was used to mesh the target body. Compared with fully integrated units, the reduced integration units use one fewer integral in each direction. Due to the large deformation in the contact area between projectile and target body, the deformation in other positions far from the contact area is relatively small, but local mesh refinement was performed on the surface contact area of the target body and along the thickness direction to improve the calculation speed while ensuring the accuracy of the simulation results. The minimum element size of the contact area between target body and projectile was set to 0.03 mm. The target model had 184049 grids in total. Throughout the entire shot peening simulation process, the total energy of the multi-shot model system remained constant at approximately 1.6 mj. Before the projectile hit the surface of the target, its kinetic energy was maximal (approximately 1.6 mj), and the internal energy of the target was 0 mj. When the contact area between projectile and target surface increased, the kinetic energy of the projectile gradually decreased, and the internal energy of the target gradually increased. Due to the distribution of the three layers of projectiles in the model, the changes in kinetic energy and internal energy of the entire model were divided into three stages. At 60 μs, the kinetic energy of the projectile was minimal, and the internal energy of the target material was maximal, which indicates that the kinetic energy of the projectile is gradually converted into the internal energy of the target body, and the projectile has begun to detach from the surface of the target body.

**Table 4:** Material parameters of the sample and projectile [21].

material	Density (g.cm^-3^)	Elastic modulus (MPa)	Poisson’s ratio	Yield strength (MPa)	Tensile strength (MPa)	Elongation
sample	7.85	190000	0.21	1512	1636	13%
projectile	7.85	210000	0.3	-	-	-

## 3. Results and discussion

### 3.1 Microstructure

**Fig 3:**
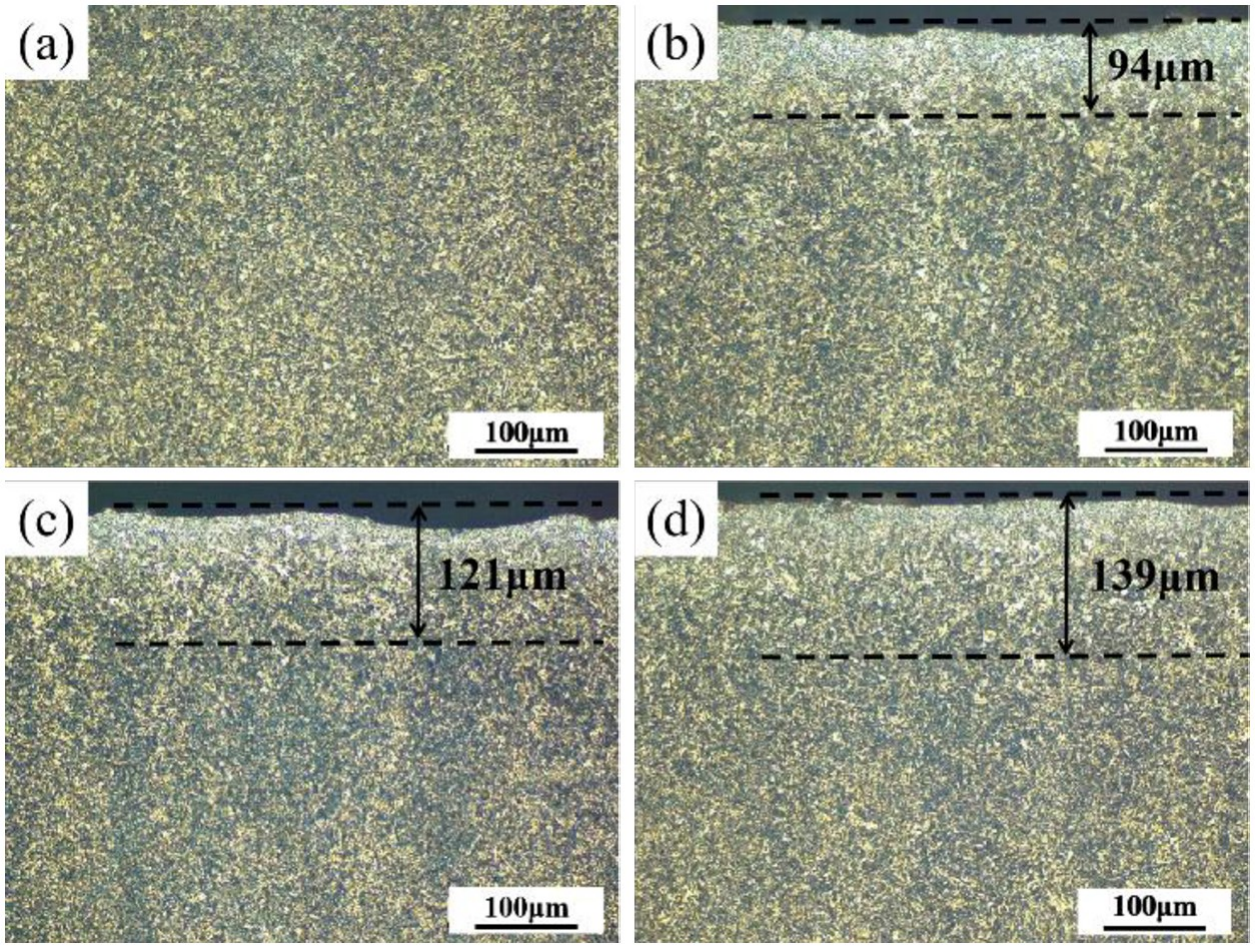
Microstructure of various samples: (a) UT, (b) SP1, (c) SP2, and (d) SP3.

Fig 3 shows the cross-sectional metallographic structures of the UT sample and shot-peened sample. The metallographic structure of all sample sections is composed of lath martensite and retained austenite. Fig 3(a) shows the metallographic structure of the UT sample, and the microstructure of the UT sample is relatively uniform overall. Fig 3b–3d show the cross-sectional metallographic structures of the SP1, SP2 and SP3 samples. Obvious grain refinement appeared on the surface of the sample after shot peening, which resulted in a gradient structure from the surface to the interior of the material. The microstructure layers of the three groups of samples were 94 μm, 121 μm and 139 μm thick. The thickness of the refined layer increased with increasing diameter of the particle. The reason is that increasing the particle diameter is equivalent to increasing the particle mass, so the particle has greater kinetic energy to hit the sample surface, which leads to more severe plastic deformation on the sample surface.

### 3.2 Microhardness

**Fig 4:**
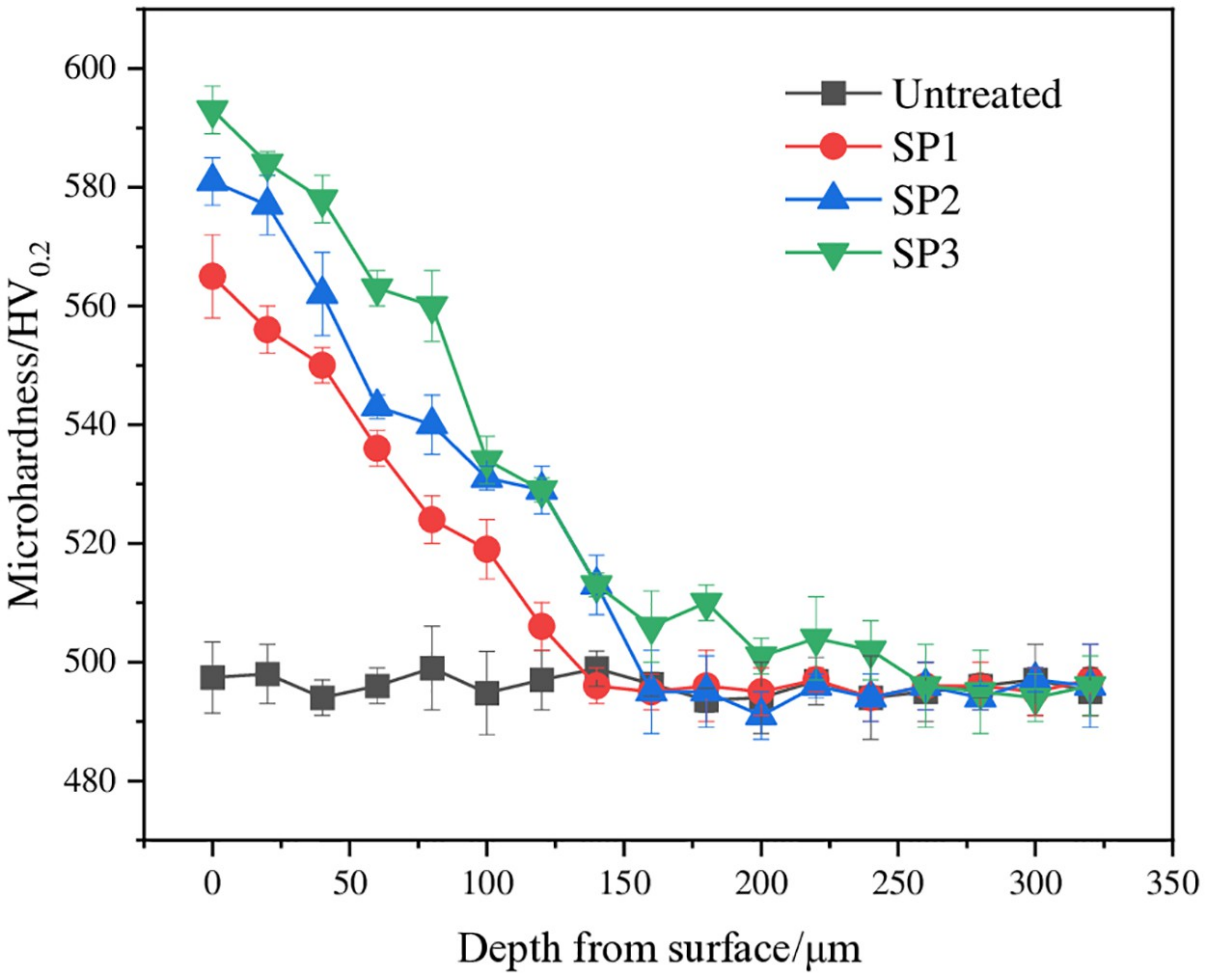
Microhardness of various samples.

Fig 4 shows the variation curves of the microhardness of various samples with depth. The figure shows that the hardness of the UT sample was approximately 500 HV_0.2_. The microhardness of the shot-peened sample tended to decrease from the material surface to the interior of the matrix until it was basically consistent with the hardness value of the matrix. The surface hardness of the shot-peened sample increased with increasing particle diameter, and the maximum hardness values were 563.7 HV_0.2_, 581.9 HV_0.2_ and 592.3 HV_0.2_, which were 14.3%, 18.0% and 20.1% greater than those of the UT sample, respectively. The maximum surface hardness was obtained when the particle diameter was 0.8 mm. In addition, the magnitude of the increase in surface hardness of the sample decreased with increasing particle diameter. When the material undergoes severe plastic deformation, the grain refinement dominated by dislocation propagation and the grain coarsening dominated by grain boundary migration in the microstructure of the material are in balance; finally, the limit grain size is reached, and the material continues to deform. The hardness cannot be improved at this time [22, 23]. Therefore, in an appropriate range, the hardness of a sample can be increased by increasing the diameter of the pellets.

### 3.3 Residual stress

**Fig 5:**
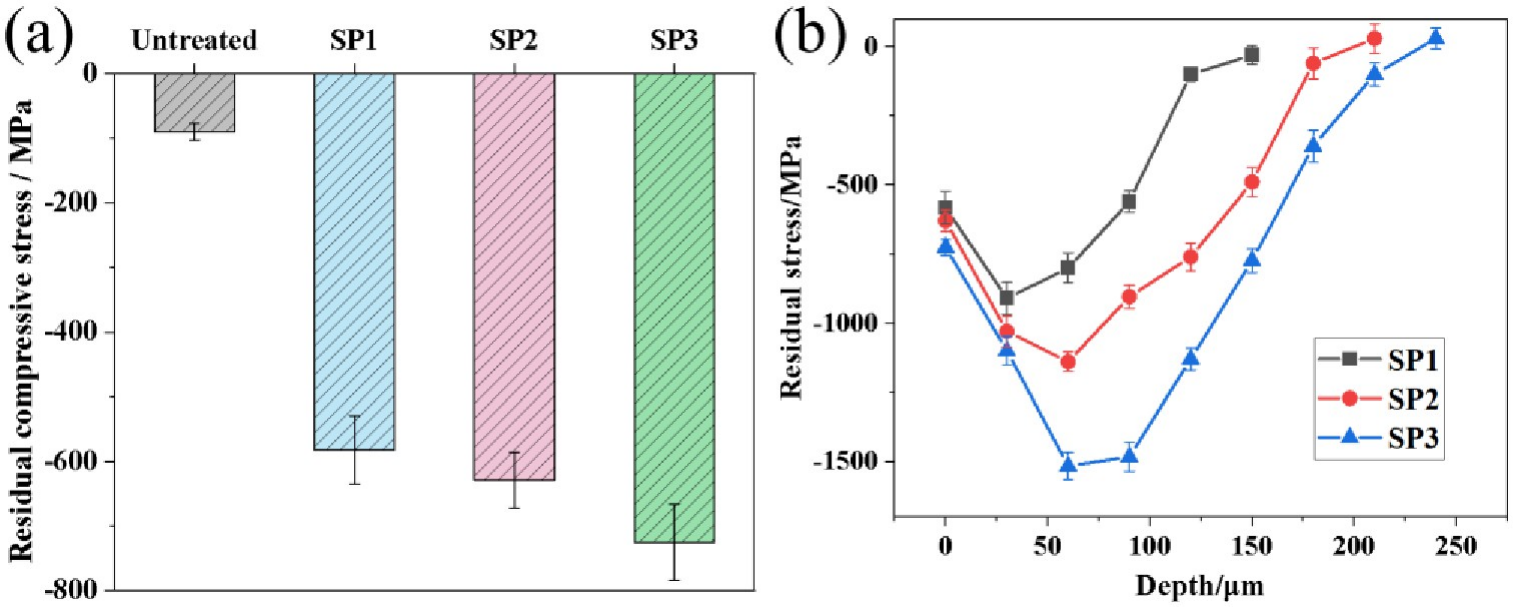
Residual stress of various samples: (a) surface residual stress and (b) residual stress in the depth direction.

The residual stress is an important indicator to evaluate the shot peening process parameters. Shot peening causes severe plastic deformation on the surface of a sample, which results in grain refinement, lattice distortion, and residual compressive stress [24]. Fig 5 shows the surface residual compressive stress of various samples. The residual compressive stress on the UT sample surface was -90 MPa due to the combined effect of heat treatment and mechanical processing technology to introduce less residual compressive stress on the material surface. When the diameter of the projectile increased, the surface residual compressive stresses of the shot-peened sample significantly increased to -582 MPa, -629 MPa, and -725 MPa, which are 6.5, 7.0, and 8.1 times greater than those of the UT sample, respectively. Fig 5(b) shows the distribution of residual stress at depth. The maximum residual stress occurred in the subsurface layer. The maximum residual stresses occurred at -30 μm, -60 μm, and -60 μm and were -907 MPa, -1141 MPa, and -1518 MPa, respectively. The depths of the residual stress layer were 130 μm, 180 μm, and 260 μm and increased with increasing projectile diameter. When the diameter of the particle increased, the kinetic energy of the particle increased. The metal grains on the surface of the sample absorbed more energy and experienced severe plastic deformation, which converted the kinetic energy of the particle into the internal potential energy of the grains and increased the residual compressive stress. The residual compressive stress on the surface layer can increase the closing force of microcracks and prevent crack propagation [25] to improve the wear resistance of the workpiece surface.

### 3.4 Surface morphology and roughness

**Fig 6:**
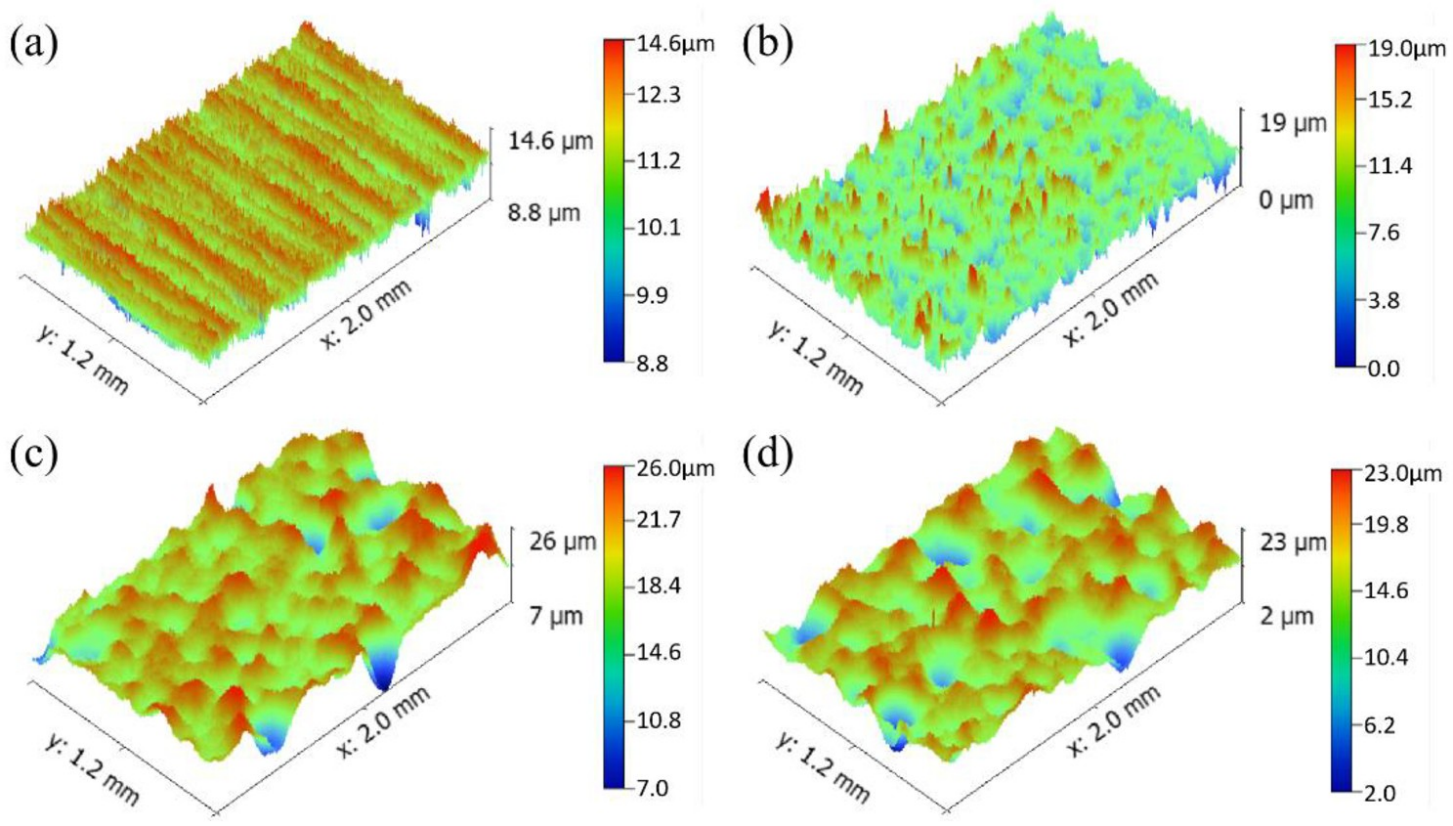
Surface 3D topography (a) UT, (b) SP1, (c) SP2, (d) SP3.

Machining will cause parallel wear marks on the surface of the material and result in tiny defects, where fatigue cracks easily initiate. After the shot peening treatment, the machining tool marks on the surface of the parts disappear, and small craters with disordered distributions are produced, which reduce the number of defects formed by machining, improve the surface properties of the parts, and help improve the wear resistance of the parts. Fig 6 shows the 3D topography of the surface of the untreated and shot-peened samples, where red represents the raised parts, and blue represents the depressed parts. After the shot-peening treatment, the topography of the sample surface was clearly observed. Fig 6(a) shows many parallel grinding marks on the surface of the UT sample, which was an obvious grinding groove shape. The surface of the material was relatively uniform without obvious fluctuations, and the height difference between protrusions and pits was 5.8 μm. Fig 6b–6d show many tiny craters on the surface of the sample in the shot peening area with large fluctuations and a typical dimple shape. The depths of the craters were 19 μm, 19 μm and 21 μm. Different shot diameters caused basically identical crater depths, but the area of the crater increased with increasing shot diameter.

**Fig 7:**
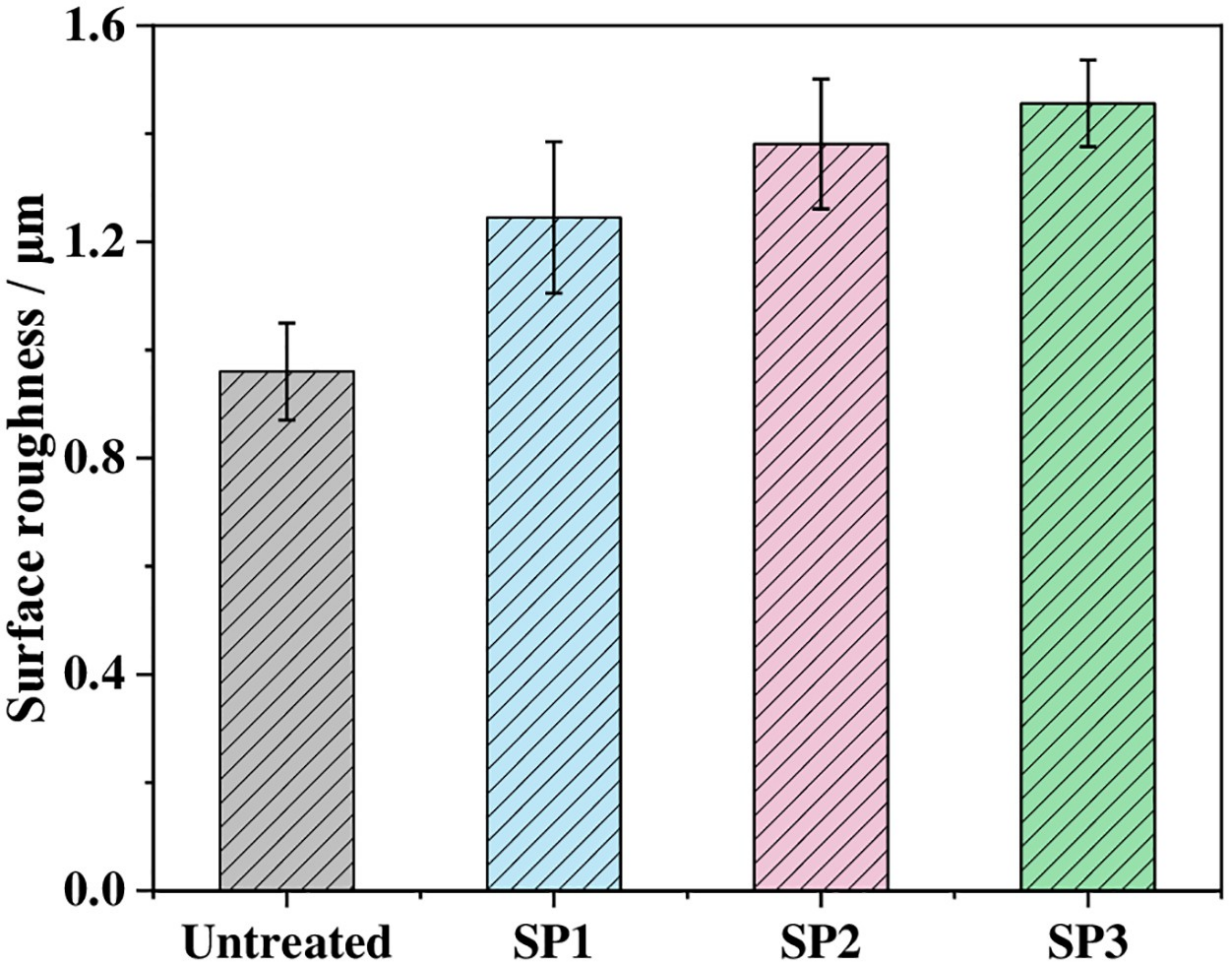
Surface roughness of various samples.

Fig 7 shows the surface roughness of the untreated and shot-peened samples. The surface roughness Ra of the UT sample was 0.960 μm. After shot peening, the surface roughness of the sample gradually increased with increasing shot diameters of 1.245 μm, 1.381 μm and 1.456 μm. Compared with those of the UT sample, they increased by 29.7%, 43.9% and 51.7%, respectively. Kovacı et al. [9] reported that an increase in shot peening intensity implies that the degree of severe plastic deformation on the surface of a sample increases, which increases the roughness, and greater surface roughness causes stress concentration. This behavior may reduce the wear resistance of the material.

### 3.5 Simulation analysis

**Fig 8:**
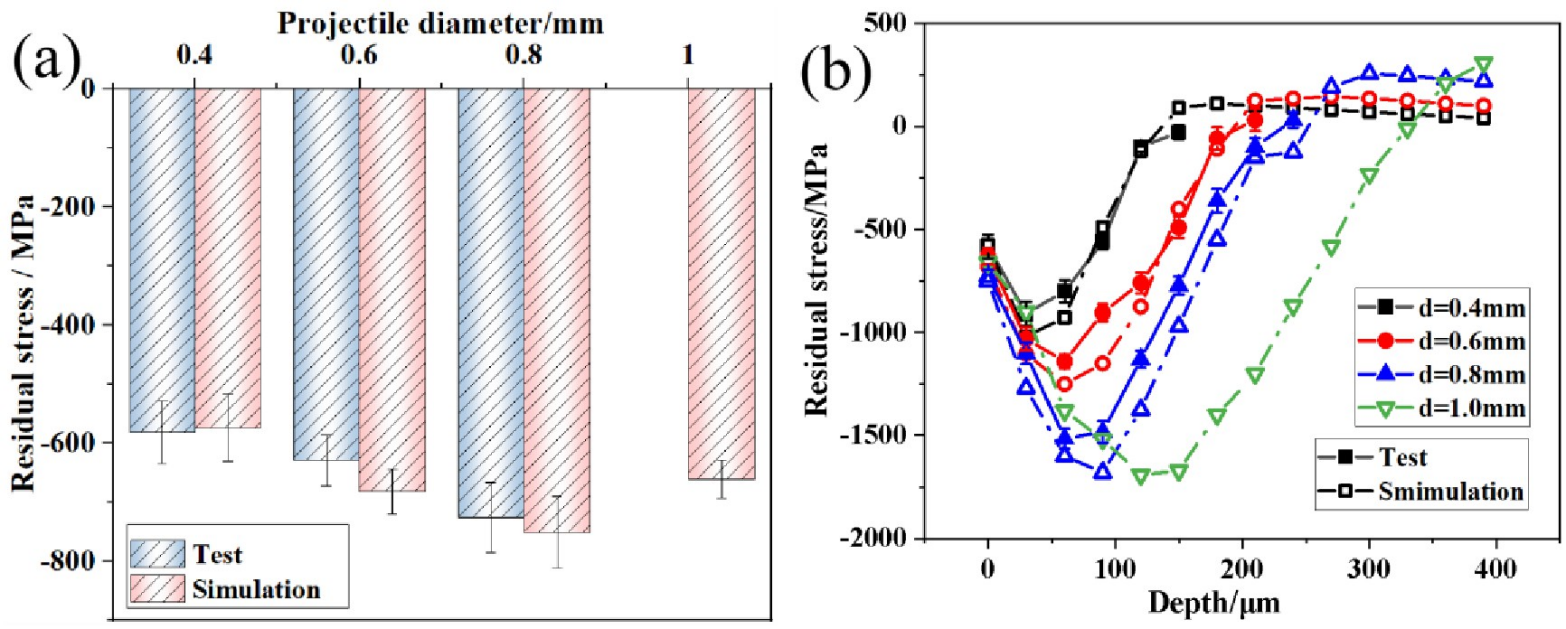
Residual stress results of the finite element model: (a) comparison between surface residual stress and test values; (b) distribution along the depth direction.

Fig 8 shows the residual stresses in the finite element model after shot peening. In Fig 8(a), the surface residual compressive stresses for the four sets of samples were -573.6 MPa, -681.3 MPa, -750.7 MPa, and -660.4 MPa. Compared with the experimental results, the error between finite element model results and test results were 1.4–7.9%. This consistency indicates the high accuracy of the model. Fig 8(b) indicates that the residual compressive stresses along the depth direction of the samples first increased and subsequently decreased. The maximum residual compressive stress was located in the subsurface of the samples, after which it transitioned into residual tensile stress. The surface residual compressive stresses first increased and subsequently decreased. The maximum residual compressive stresses, corresponding layer depths, and depth of the residual stress field sequentially increased. The maximum residual compressive stresses were -987.2 MPa, -1241.9 MPa, -1586.5 MPa, and -1609.2 MPa, whereas the residual stress field depths were 0.12 mm, 0.18 mm, 0.21 mm, and 0.33 mm, respectively. Fig 8(b) shows that the maximum residual compressive stresses of the simulation results were -987.2 MPa, -1241.9 MPa, -1586.5 MPa, and -1609.2 MPa. The depths of the residual compressive stress fields were 0.12 mm, 0.18 mm, 0.21 mm, and 0.33 mm. Fig 8(b) shows that the test values were generally lower than the simulated values. However, the residual stress variation trends calculated by the finite element model at different depths were consistent with the test values, which proves that the finite element model can accurately calculate residual stresses under different conditions. The increases in maximum residual compressive stress and layer depth in the samples are attributed to the increase in kinetic energy of the projectiles with increasing projectile diameter. This increase deepened the plastic deformation in the surface layer of the samples, which increased the maximum residual compressive stress. Additionally, the stress waves that were generated when the projectiles impacted the surface of the samples propagated deeper into the material, which increased the depth of the residual compressive stress field. Notably, when the projectile diameter increased from 0.8 mm to 1.0 mm, the increase in maximum residual compressive stress in the subsurface of the samples was not significant, whereas the surface residual compressive stress values significantly decreased. This finding indicates that in practical shot peening processes, the selection of larger projectile diameters is primarily aimed at increasing the depth of the surface residual compressive stress field.

**Fig 9:**
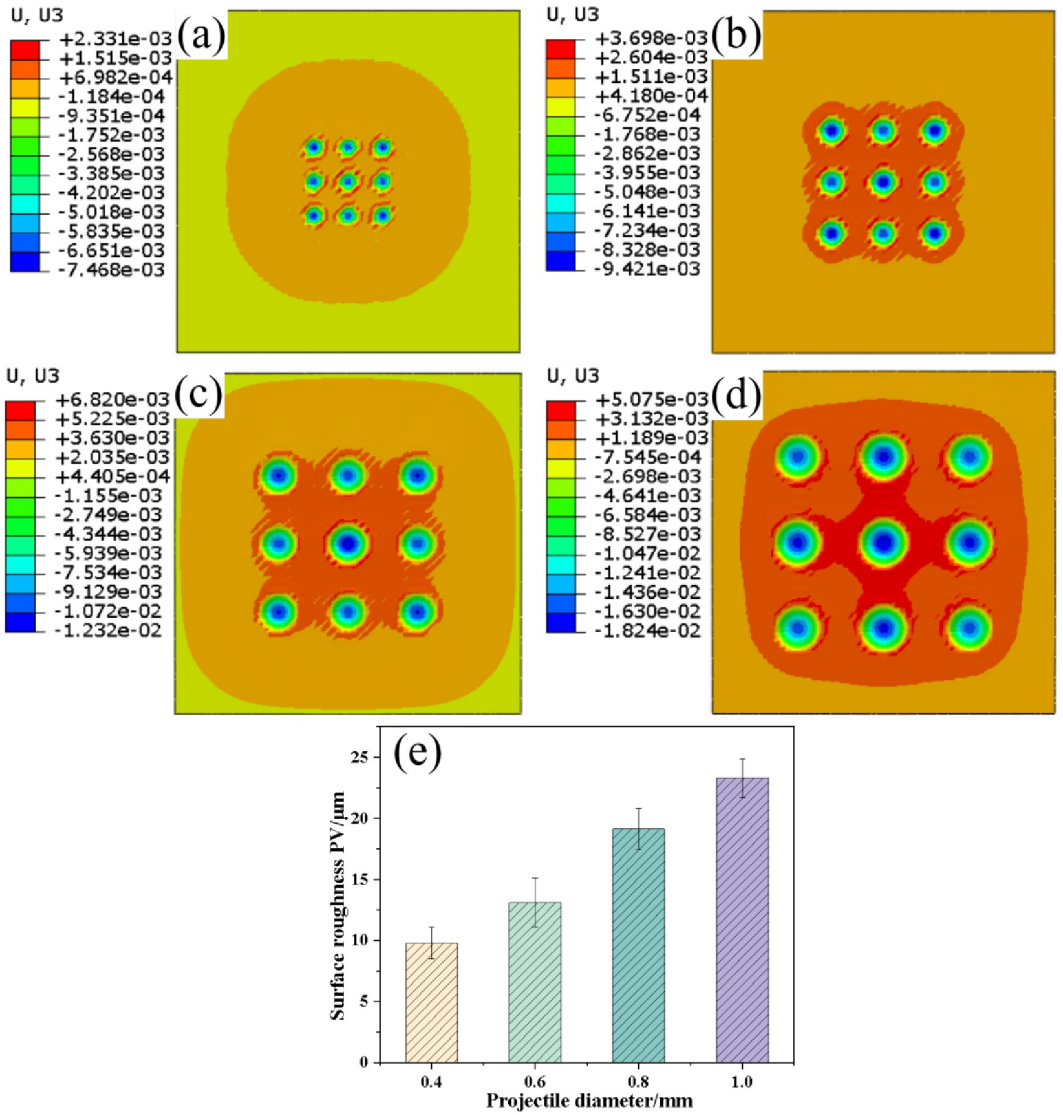
Surface displacements after shot peening in the finite element model for different projectile diameters: (a) 0.4 mm, (b) 0.6 mm, (c) 0.8 mm, (d) 1.0 mm, and (e) surface roughness PV.

Fig 9 shows the displacement contour map of the samples along the Z-axis after shot peening. With increasing projectile diameter, the range of the raised area on the target surface gradually expanded. Simultaneously, the depth of the craters, size of the craters, and height of the raised areas on the target surface significantly increased. The peaks of the target displacement were 2.3 μm, 3.7 μm, 6.8 μm, and 5.1 μm, whereas the valleys of the displacement were 7.5 μm, 9.4 μm, 12.3 μm, and 18.2 μm, respectively. This result was attributed to the increase in kinetic energy of the projectiles with larger diameters, which caused more pronounced plastic deformation in the target material. Consequently, the height difference between raised and cratered areas on the target surface increased when the projectile diameter increases while maintaining constant shot peening air pressure and coverage.

In this study, the difference between peaks and valleys (PVs) was used to represent the surface roughness of the target material after shot peening. The highest point along the Z-axis on the target surface represents the peak, whereas the lowest point represents the valley. By combining the numerical results from the displacement contour distribution in Fig 9, this method was used to quantify the surface roughness of the target material in shot peening simulations. Fig 9(e) shows the surface roughness (PV) of the target material after shot peening with different projectile diameters. When the projectile diameter increases, the surface roughness PV of the target material sequentially increased to 9.80 μm, 13.12 μm, 19.14 μm, and 23.32 μm for projectile diameters of 0.4 mm, 0.6 mm, 0.8 mm, and 1.0 mm, respectively. Compared with a projectile diameter of 0.4 mm, the surface roughness increased by 2.4 times when the projectile diameter was 1.0 mm. This result was primarily attributed to the increase in kinetic energy of the projectiles with larger diameters, which enhanced the impact forces on the target surface and the surface roughness. The variation in surface roughness in the finite element model is closely consistent with the experimental results of shot peening.

## 4 Friction and wear behavior

### 4.1 Friction coefficient

**Fig 10:**
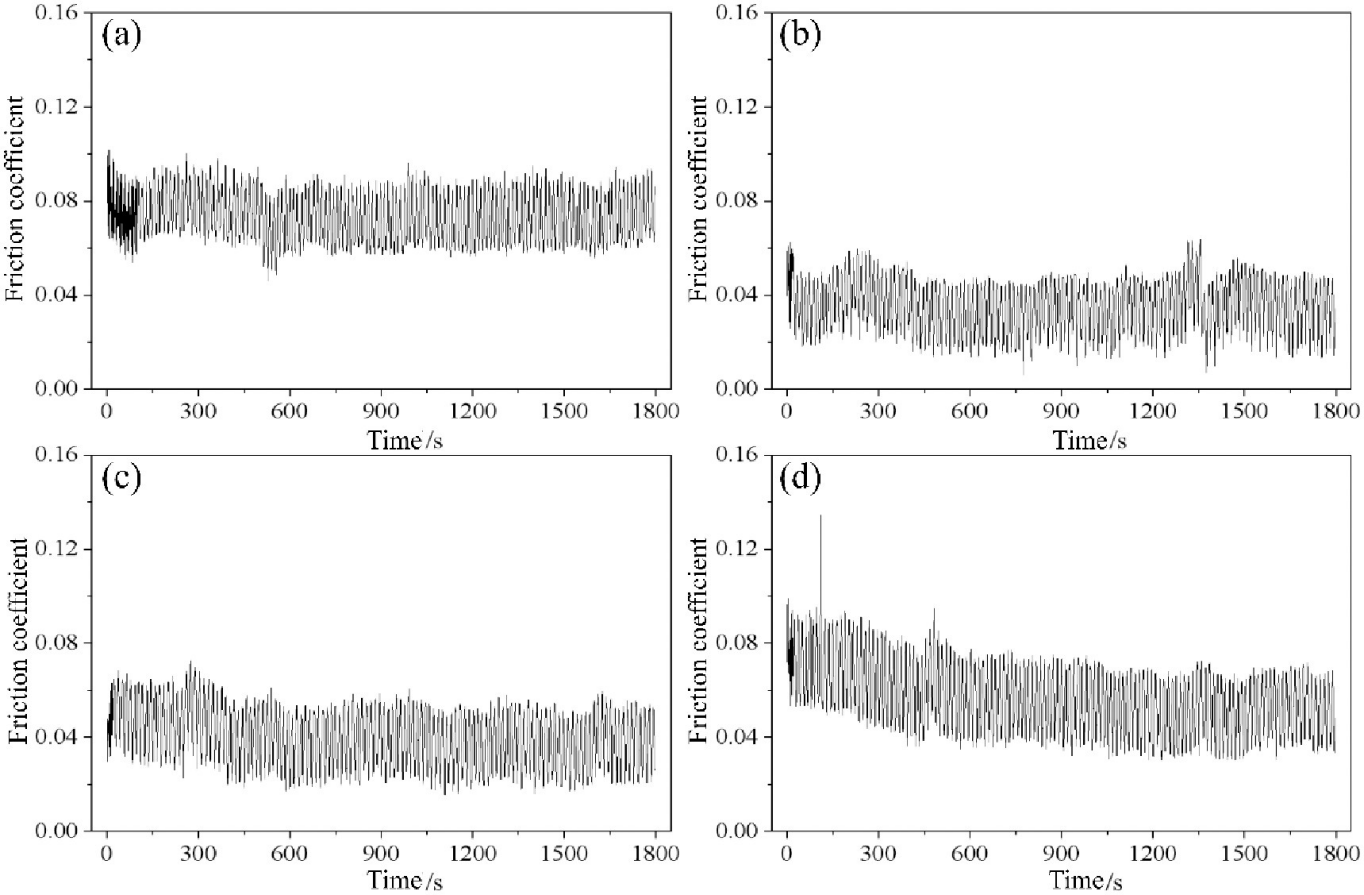
Friction coefficients of various samples: (a) UT, (b) SP1, (c) SP2, and (d) SP3.

The friction coefficient is a comprehensive reflection of many factors such as the friction pair material, surface morphology, processing, heat treatment, lubrication state and test conditions (pressure, temperature and speed, etc.) [26]. Fig 10 shows the sliding friction coefficients of the UT samples and SP samples. The friction coefficients of the four groups of samples were relatively small, and the overall change was relatively stable, which was attributed to the lubricating effect of the anti-wear hydraulic oil. The UT sample had a more stable friction coefficient than the SP sample. During the sliding wear test, the ball pin and original disk sample maintained a roughly stable friction state. The friction coefficient of the original disk sample fluctuates at 0.06–0.09 with relatively high values. Compared with the UT sample, sample SP1 had a smaller friction coefficient of 0.02–0.05. The friction coefficient of sample SP2 decreased with longer wear time, but compared with SP1, the friction coefficient slightly increased and fluctuated between 0.03 and 0.06. SP3 had a similar friction coefficient to SP2, and the value was 0.04–0.08. The overall value of SP3 was smaller than that of the UT sample and larger than those of SP1 and SP2.

**Fig 11:**
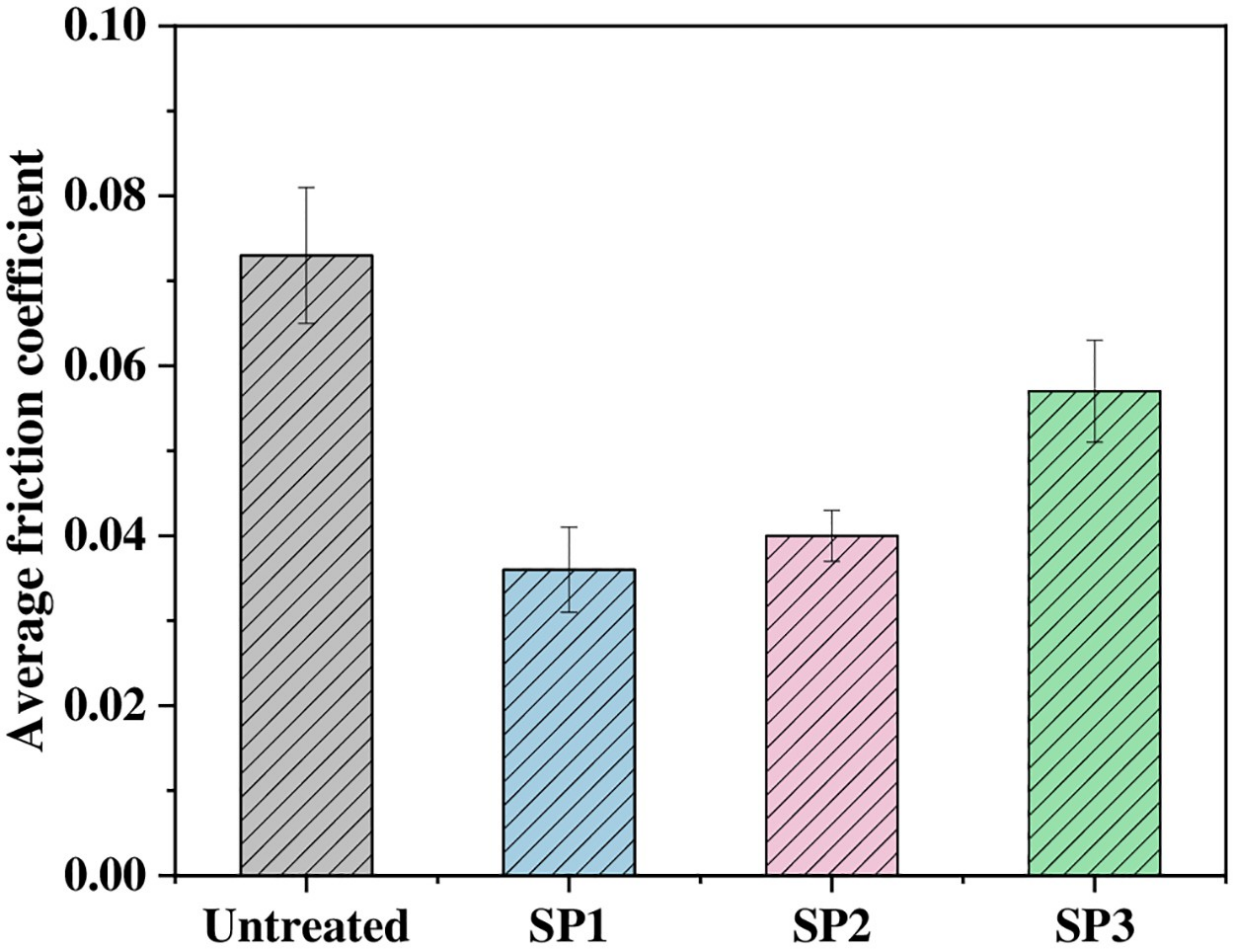
Average coefficient of friction of various samples.

Fig 11 shows the average sliding friction coefficients of various samples. The average coefficient of friction of the UT sample was 0.073. With increasing shot peening intensity, the average friction coefficients of the shot peened samples sequentially increased to 0.036, 0.040 and 0.057, which were 50.7%, 45.2% and 21.9% lower than those of the UT samples, respectively. These findings indicate that shot peening can effectively reduce the friction coefficient of a sample; the reason is that the shot peening treatment increases the surface hardness of the sample, refines the grain size, and introduces a residual compressive stress field on the material surface, which can offset the frictional tensile stress borne by the sample and reduce the coefficient of friction. In addition, the shot peening treatment forms a tiny crater shape on the surface of the sample, which becomes a micro pool to store lubricating oil, enhances the hydrodynamic lubrication effect, reduces the contact stress and reduces the friction coefficient [18]. The coefficient of friction increases with increasing shot peening intensity mainly because when the shot peening intensity increases, the surface roughness of the sample increases, and higher contact stress is easily generated at the asperities on the sample surface. When the contact stress is greater than the bearing capacity of the shot peening layer of the sample, the surface material of the sample will "collapse" and peel off, boundary lubrication wear changes to adhesive wear with the matrix, and the friction coefficient consequently increases [27].

### 4.2 Wear behavior

**Fig 12:**
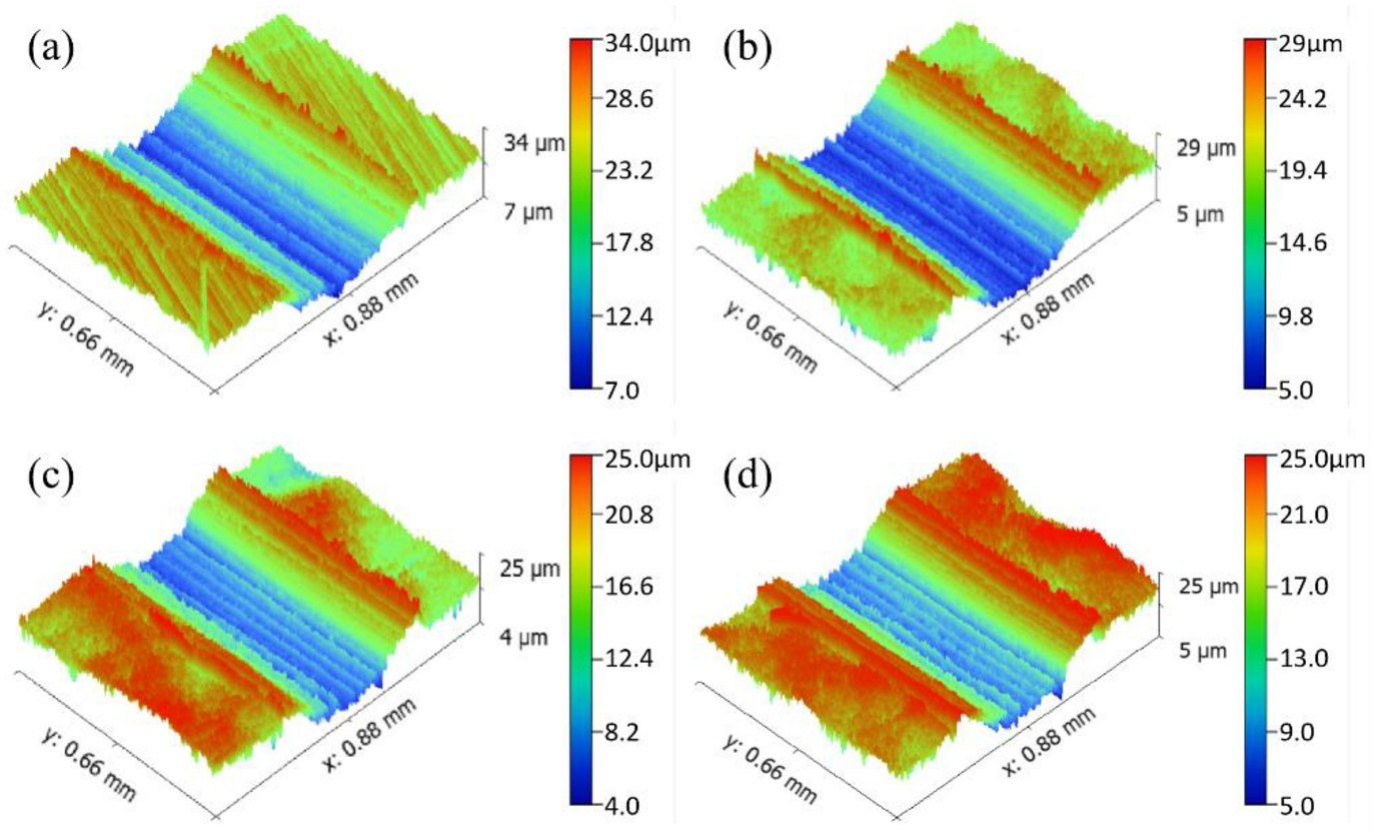
Wear morphology: (a) UT, (b) SP1, (c) SP2, and (d) SP3.

Fig 12 shows the sliding wear morphology of various samples. The plow effect at the bottom of the wear scar of the UT sample was obvious, and the depth of the wear scar was 27 μm. A slight bulge appeared at the edge of the wear scar because the edge area of the sample was squeezed by the ball stud to form a bulge. Fig 12b–12d show that the bottom of the wear scar of the SP1 sample was relatively flat, the furrow effect was weak, and the depth of the wear scar was 24 μm. The wear scars of samples SP2 and SP3 were 21 μm and 20 μm deep, respectively. When the shot peening intensity increased, the wear scar depth of the sample sequentially decreased, which indicates that shot peening treatment can effectively reduce the sliding wear scar depth of the sample.

### 4.3 Wear mechanism

**Fig 13:**
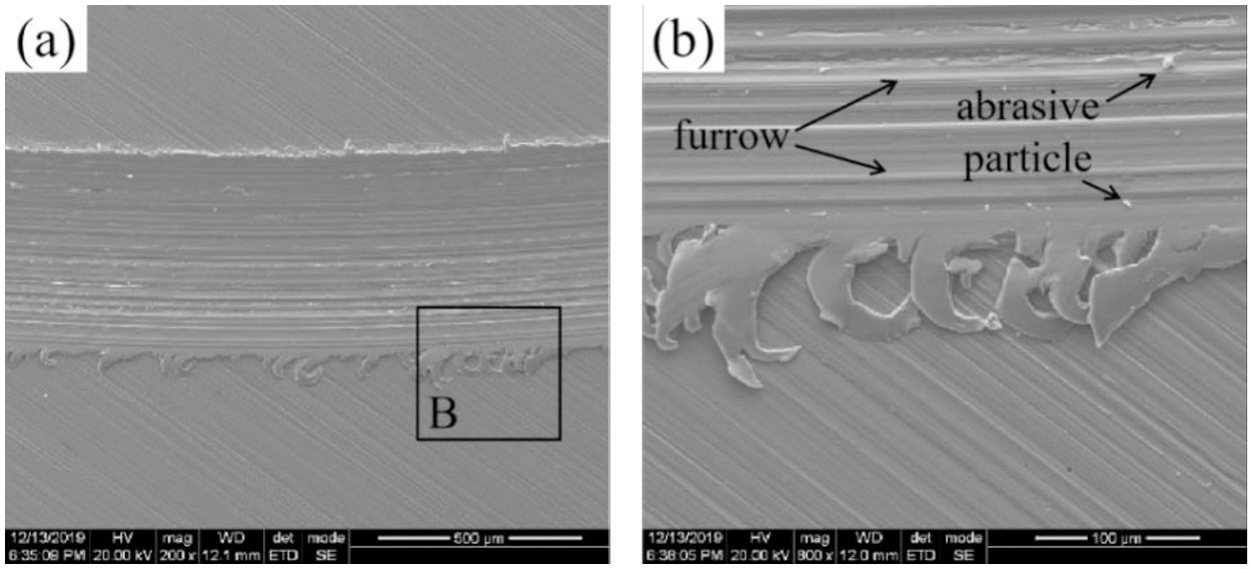
Wear morphology of the UT sample.

Fig 13 shows the sliding wear morphology of the UT sample. Severe metal microcutting phenomena appeared on the surface of the UT sample, and the wear marks on the surface were wider and deeper, which were obvious furrow characteristics. A small amount of white speckled hard particles attached to the surface, and the particle size was small, which indicates typical abrasive wear. Since the UT sample was not strengthened, the surface hardness of the sample was low, the surface roughness was small, and an oil film could not be formed, which increased the relative motion resistance of the ball stud and disc samples. Thus, the friction coefficient and wear volume were large. A large amount of shear debris formed during the wear process at the edge of the wear scar, as shown in Fig 13(b). This finding reveals that the part of the material in the contact area of the sample with the ball stud pin has undergone severe shear deformation because the UT sample has lower strength, lower hardness, and a weaker ability to resist plastic deformation.

**Fig 14:**
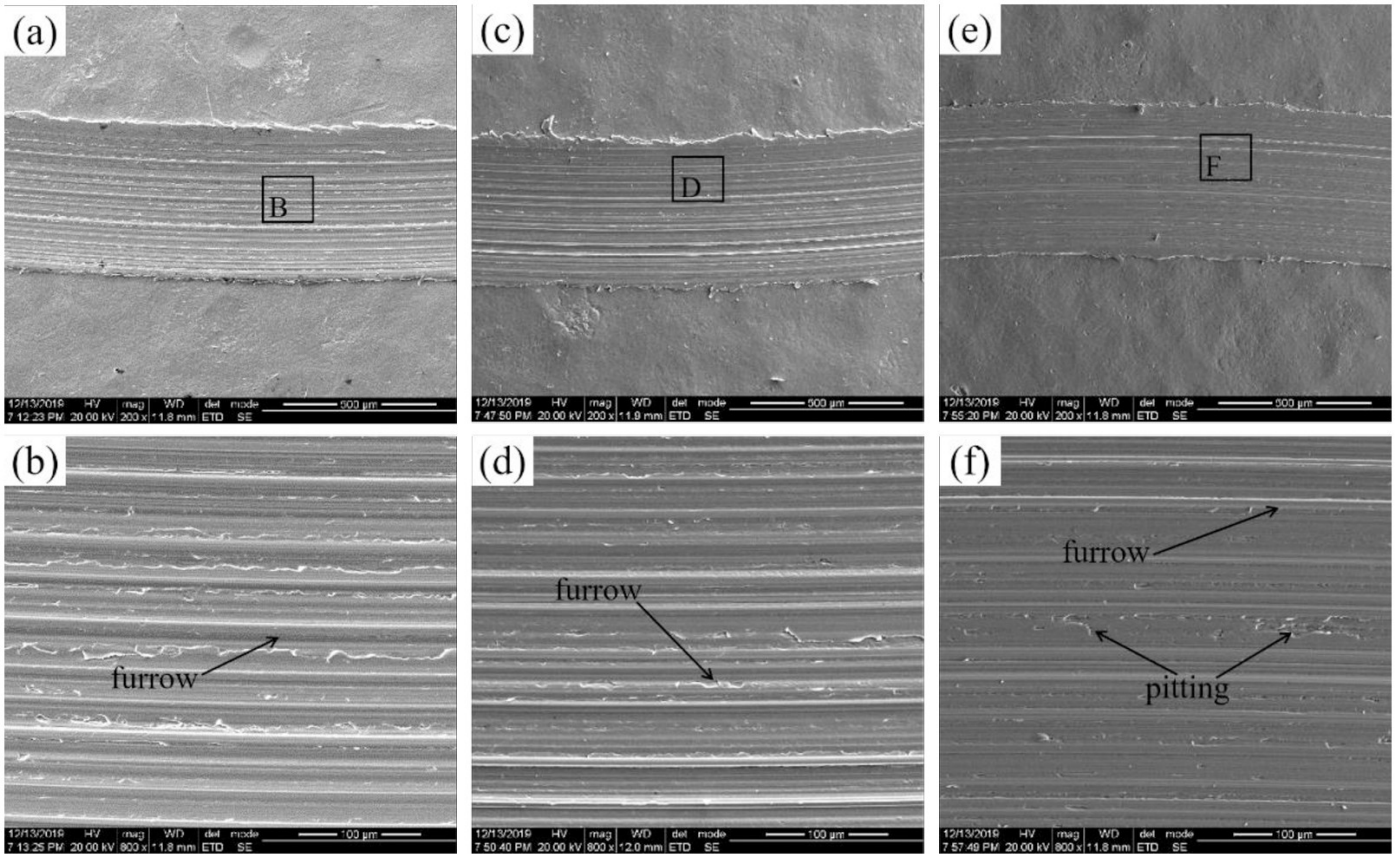
Wear morphology of the SP samples: (a) (b) SP1, (c) (d) SP2, and (e)(f) SP3.

Fig 14 shows the sliding wear morphology of the shot peened samples at room temperature. The wear morphologies of all three groups of shot peened samples showed the characteristics of abrasive wear, and the wear mechanism was mainly abrasive wear. Compared with those of the UT sample, the wear scars of the shot-peened sample were narrower and shallower, which indicates that the wear state of the material was relatively stable, and the sample after the shot-peening treatment had excellent wear resistance. The reason is that the material formed a residual compressive stress field and a hardened layer at a certain depth under the action of shot peening, which increased the fatigue resistance and plastic deformation resistance, so the wear resistance of the material improved [28]. In addition, after the shot peening treatment, the surface of the sample was evenly distributed with tiny craters. Although craters increase the surface roughness, they can store lubricating oil. In the sliding wear test, the oil film easily formed, which effectively reduced the friction and wear on the material surface. Therefore, the anti-wear performance of the shot peened sample improves.

Fig 14 shows that when the shot peening intensity increased, the white spot-like hard particles that appeared in the wear morphology of the sample decreased, and the width and depth of the surface wear marks decreased, which indicates that the wear resistance of the sample increased. An increase in surface hardness can enhance the plastic deformation resistance of the material surface, and the residual compressive stress can inhibit the initiation and propagation of cracks on the material surface [29]. The SP3 sample exhibited the best sliding wear resistance because it had the greatest surface hardness and residual compressive stress. Therefore, shot peening can significantly improve the wear resistance of materials by improving their surface properties, among which the surface hardness and residual compressive stress are the main factors.

## 5 Conclusion

In this work, the effects of shot peening experiments with different diameters on the sliding wear properties of 25CrNi2MoV steel were compared. The conclusions are as follows:

1. Obvious grain refinement can be obtained on the material surface after shot peening. With increasing shot diameter, the depth of the grain refinement layer significantly increased to a maximum of 139 μm.
2. With increasing shot peening intensity, the hardness and residual stress of the sample surface significantly increased to maximum values of 592.3 HV_0.2_ and -725 MPa, respectively.
3. The error of the shot peening finite element model was 1.4–7.9% with residual stress field depths of 0.12 mm, 0.18 mm, 0.21 mm, and 0.33 mm. When the projectile diameter increased to 0.8 mm, the residual stress in the sample did not continue to increase.
4. Shot peening can significantly improve the sliding wear properties of materials. Under the action of high hardness and high residual stress after shot peening, the wear resistance of materials significantly improved, and the wear volume significantly decreased.

## Supporting information

S1 File https://doi.org/10.5061/dryad.n2z34tn55. (DOCX)
