# Supplementary material for: Study on the effects of the shot peening intensity on the microstructure, friction and wear properties of high-strength steel
Source: PLoS One. 2024 Dec 19;19(12):e0314561. doi: 10.1371/journal.pone.0314561 (PMC11658633; doi:10.1371/journal.pone.0314561)
Supplement: S1 File — (DOCX) [file pone.0314561.s001.docx]

**Database URL：**[**https://doi.org/10.5061/dryad.n2z34tn55**](https://doi.org/10.5061/dryad.n2z34tn55)

**All data has been uploaded to the database and can be viewed by everyone.**
